# Uric acid levels and risk of cognitive impairment: Dose-response meta-analysis of prospective cohort studies

**DOI:** 10.1371/journal.pone.0293832

**Published:** 2023-11-02

**Authors:** Qianqian Liu, Min Peng, Tiantian Yang, Guomin Si

**Affiliations:** 1 School of Traditional Chinese Medicine, Shandong University of Traditional Chinese Medicine, Jinan, China; 2 Department of Chinese and Western Medicine, Shandong Provincial Hospital Affiliated to Shandong First Medical University, Jinan, China; Universiti Sains Malaysia, MALAYSIA

## Abstract

**Purpose:**

Studying the effects of uric acid levels on cognitive function and quantifying the dose-response relationship.

**Methods:**

Based on PubMed and Embase search terms, we identified prospective cohort studies that included blood uric acid as a risk factor and cognitive impairment as a result up to September 2022. We extracted pooled relative risks (RRs) and corresponding 95% confidence intervals (CIs).

**Results:**

Nine reports (including 488,915 participants and 5516 cognitive impairment cases) with median follow-up of 8.8–22 years were eligible for analyses. Compared with lowest category of blood uric acid concentration, the combined RR of cognitive impairment events in the highest classification was 0.81 (95% CI: 0.70–0.92, P < 0.001). Dose-response analysis of eight reports (including 484,297 participants and 5059 cognitive impairment cases) showed that there was no evidence of a curvilinear relationship between blood uric acid levels and cognitive impairment (P = 0.51 for nonlinear relationship). The summary RR of cognitive impairment for an increase of 1 mg/dL blood uric acid level was 0.98 (95% CI: 0.95–1.00; linear trend P = 0.07, I^2^ = 67.1%, heterogeneity P < 0.05). There was also a linear negative association between blood uric acid levels and cognitive impairment risk in the male subgroup analysis (RR = 0.97, 95% CI: 0.95–0.99, P < 0.05).

**Conclusion:**

Levels of blood uric acid are not related to risk of cognitive impairment. A subgroup analysis shows that the rise in blood uric acid levels in the male population is related to a decreased risk of cognitive impairment. These results need to be confirmed by further studies.

## 1 Introduction

Cognitive impairment is an umbrella term for impairment of one or more cognitive domains which may be influenced by various causes and may range from mild cognitive impairment (MCI) to different stages of dementia [1, 2]. With the increase in population aging, cognitive impairment has become a global public health concern, causing serious social and economic losses [3, 4]. MCI is estimated to affect approximately 25% of people aged 80–84 years, with a cumulative incidence of dementia of 14.9% in people aged 65 years and older at 2 years of follow-up [5]. An estimated 55 million people were living with dementia in 2019, it is predicted that by 2050, this number will reach 139 million, according to the updated data from the World Health Organization (WHO) [6]. With the full implementation of the seven priority action areas of the WHO Global Action Plan on the Public Health Response to Dementia, more attention has been paid to prevent disease progression and delay its progression by controlling modifiable risk factors [1, 7, 8]. Among the known risk factors, blood uric acid (UA) has attracted particular attention.

The body produces UA through purine metabolism and it has both oxidation-promoting and anti-oxidant properties. It has been found that UA may be involved in mechanisms such as oxidative stress and inflammatory responses associated with cognitive impairment and acts as a double-edged sword for cognitive function in the brain [9]. Some observational studies suggest that high UA levels may have deleterious effects on brain health, including the development of cerebral small vessel disease and cognitive decline [10, 11]. However, studies have also indicated that serum UA plays a neuroprotective role in the diseases of Alzheimer’s and Parkinson’s and that low blood UA levels are not only associated with faster disease progression, but are also indicative of malnutrition [12–15]. Appropriate increases in UA levels within the normal range can instead delay the onset and progression of cognitive impairment [16]. Notably, many vascular risk factors and diseases have also been associated with UA, which may predispose people to cognitive impairment [2]. This may also, to some extent, alter the relations between UA levels and poor cognitive outcomes. In addition, recent studies suggest that the impact of UA levels on cognitive function may vary according to gender and dementia subtype, but with variable results [17, 18].

The above cumulative findings develop new approaches to identifying and treating cognitive impairment risk factors and highlight the potential for blood UA management in the prevention of cognitive impairment. However, the correlation between blood UA and cognitive impairment remains controversial, although extensive research had been utilized to explore the above relationship. Furthermore, studies have examined whether blood UA is associated with cognitive impairment in published systematic reviews [19, 20], but not specifically within prospective cohort studies, and no relevant dose-response analyses have been evaluated. Therefore, we sought to quantify the relationship between blood UA levels and cognitive impairment by conducting a dose-response meta-analysis of prospective cohort studies.

## 2 Material and methods

### 2.1 Search strategy

From inception until September 2022, we searched PubMed and Embase for prospective cohort studies investigating UA levels and cognitive impairment (including MCI and dementia of all types). The meta-analysis was prospectively registered in INPLASY (2022100111). Both MESH subject headings and free terms were employed in the search (S1 and S2 Tables). Furthermore, a thorough review of the references of the relevant original articles was also undertaken to uncover further related studies. It was not restricted in any way in terms of language. Two researchers independently scrutinized all the retrieved articles and preliminary eligibility was carefully reviewed in accordance with titles, abstracts, and text in full when necessary. PRISMA (Preferred Reporting Items for Systematic Reviews and Meta-Analyses) statement was followed in our study [21].

### 2.2 Study selection

The included studies must meet the following criteria: 1) Prospective cohort studies; 2) Cognitive impairment (including all types of dementia and MCI) as a specific outcome, based on certain standardized diagnostic criteria (Table 1); 3) The investigators classified UA concentrations into at least three categories and provided relative risks (RRs) with 95% confidence intervals (CIs) for each. Furthermore, we excluded studies that lacked sufficient data, reviews, editorials, and non-human studies. Studies that were reported more than once were compiled based on the results with the longest follow-up.

**Table 1:** Assessment of cognitive function in included studies.

Author	Year	Endpoint	Case ascertainment	Diagnostic criteria	Cognition scale
Euser et al	2009	Dementia	Confirmed by a panel consisting of a neurologist, a neuro-psychologist and a research physician	DSM-III	MMSE, the Letter Digit Substitution Task, the Word Fluency Test, the Stroop test
Latourte et al	2018	Dementia	Confirmed by psychologist, neurologist and physician	DSM-IV, NINCDS-ADRDA, NINDS- AIREN	MMSE, Isaacs Set Test
Scheepers et al (women)	2019	Dementia	Confirmed by hospital register, medical records, clinical examination, close informant interview	DSM-III, ICD 8–10, NINCDS-ADRDA, NINDS- AIREN	Psycho-pathological Rating Scale, Gottfries-Brane-Steen Scale, the Mini Mental State Examination, the Alzheimer’s Disease Assessment Scale, and the Clinical Dementia Rating
Alam et al (men)	2020	Dementia	Confirmed by medical records, informant interview, telephone or death certificate	NIA-AA	The Delayed Word Recall Test (DWRT), Digit Symbol Substitution Test (DSST), and Word Fluency Test (WFT)
Alam et al (women)	2020	Dementia	Same as above	NIA-AA	The Delayed Word Recall Test (DWRT), Digit Symbol Substitution Test (DSST), and Word Fluency Test (WFT)
Cao et al (men)	2020	Dementia	Confirmed by medical records	ICD 10	Not reported
Cao et al (women)	2020	Dementia	Same as above	ICD 10	Not reported
Chen et al (men)	2021	MCI	Confirmed by face to face interview	MMSE < 24	MMSE
Chen et al (women)	2021	MCI	Same as above	MMSE < 24	MMSE

**Abbreviations**: MCI: Mild cognitive impairment; MMSE: Mini-Mental State Examination; ICD 8–10: The International Classification of Diseases, Eighth to Tenth Revision; DSM III/ IV: the Diagnostic and Statistical Manual of Mental Disorders, Third/ Fourth Edition; NINCDS-ADRDA: National Institute of Neurological and Communicative Disorders and Stroke-Alzheimer’s Disease and Related Disorders Association; NINDS-AIREN: National Institute of Neurological Disorders and the Stroke-Association Internationale pour la Recherche et l’ Enseignement en Neurosciences; NIA-AA: the National Institute on Aging and Alzheimer’s Association.

### 2.3 Data extraction and quality evaluation

Two authors (QQL and TTY) independently extracted data from each study using standard extraction formats. Each study was analyzed for the following factors: author, publication year, study name, study location, gender and age of participants, diagnostic criteria, size of the sample (number of participants and incidents), UA levels, cognitive impairment (endpoint), follow-up year and RRs (95% CIs) for different UA levels.

Studies were evaluated according to Newcastle-Ottawa quality criteria [22]. Studies with quality scores of 0 to 3, 4 to 6, and 7 to 9 were considered low quality, moderate quality, and high quality, respectively. When the study had multiple adjusted models, we extracted the model that reflected the most adjustment for potential confounders. To resolve differences by consensus, the review process was guided by group consensus and a third reviewer was consulted (MP).

### 2.4 Statistical analysis

In this study, the hazard ratios were considered equivalent to the RRs, and the RRs with 95% CI were considered to be the effect sizes of all studies. Any outcomes stratified by gender were considered to be two separate reports[23]. A dose-response relationship was investigated by using the generalized least squares regression method of Greenland and Longnecker and Orsini and colleagues [24, 25]. UA, number of reported cases, total participants, and RRs with 95% CIs were extracted for each stratum according to this method.

The RR corresponded to the mean/median of the UA level categories for each stratum in this meta-analysis. If they were not accounted for, the mean of the lower and upper limits per category was used as the dose [26]. If the highest/lowest UA level category was open, in order to calculate the midpoint, half the width of the adjacent categories were added (highest category) and subtracted (lowest category) [26]. If no numbers are available for cases or non-cases per category, the method of Chêne et al was used to supply an approximation based on cases in total and RR for per category [27, 28]. In addition, we assessed potential curvilinear relationships between UA levels and cognitive impairment using four-node restricted cubic splines at the 5%, 35%, 65% and 95% percentile distributions.

Statistical heterogeneity between studies was assessed by the I^2^ test. A low value of 25%, a medium value of 50%, and a high value of 75% were considered [29]. When heterogeneity was negligible, we used a fixed-effect model (Mantel–Haenszel method) and when it was significant, we used a random-effect model (DerSimonian and Laird method). Overall effects were examined using forest plots. Publication bias was assessed using Egger’s regression asymmetry test and Begg’s test. Stratified analyses were also conducted based on gender, age, location of the study, the length of follow-up, the number of participants, dementia type, and diabetes status. Stata version 13.0 of the software (Stata Corp, College Station, TX, USA) was used to perform all statistical analyses. Statistical significance was determined by P < 0.05.

## 3 Results

### 3.1 Literature search

**Fig 1:**
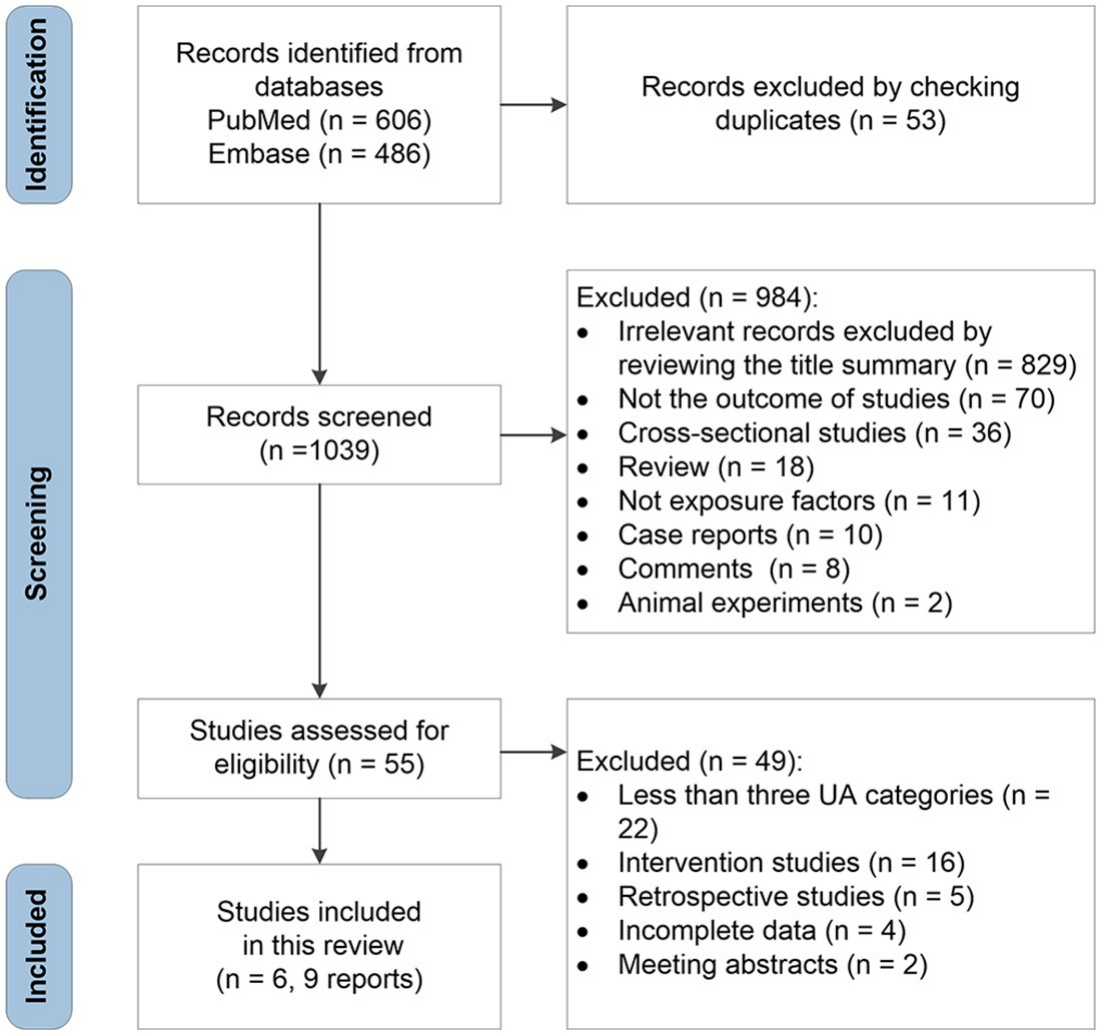
The flow diagram of study selection.

The results of the literature search and selection are shown in Fig 1. We retrieved 606 articles in PubMed and 486 articles in Embase before September 30th, 2022. After excluding duplicate papers (n = 53) and studies that failed to satisfy the inclusion criteria (n = 984), the remaining 55 articles appeared relevant to this meta-analysis. Twenty-two articles published were excluded due to less than three UA categories after reviewing these publications in their entirety. We also excluded 22 studies because they were interventional or retrospective studies. Due to insufficient data, four articles were excluded. Another two articles were excluded because they were meeting abstracts. Three of the six articles examined men and women separately in the final meta-analysis. Overall, this meta-analysis included six articles and nine independent reports. We compared the blood UA levels of the highest and lowest categories based on nine studies, and in eight studies, the impact of every 1 mg/dL of blood UA level was analyzed.

### 3.2 Study characteristics

Tables 1–4 show extracted information from the included studies. A median follow-up of 8.8 to 22 years was provided for 488,915 participants with 5516 cognitive impairments. Of the total cohort, four studies were conducted in Europe and the others were from China and the United States [30–35]. The research quality evaluation results (scores 0–9) of all studies were ≥7 (high quality), with an average score of 7.2 (Table 5).

**Table 2:** Characteristics of participants in included studies of UA in relation to risk of cognitive impairment.

Author	Year	Study name	Country	Population	Men (%)	Age at baseline (years)	Number of parti-cipants	Num-ber of cases	Foll-ow up (year)
Euser et al	2009	Rotter-dam Study	Netherl-and	All inhabitants of Ommoord	39	69.4	4618	457	11.1
Latourte et al	2018	3C-Dijon	French	Selected from the city electoral rolls	38	72.4	1598	110	10.1
Scheepers et al (women)	2019	The study of women in Gothe-nburg 1968–1969	Sweden	Derived from the Swedish census registration system	0	47.3	1447	320	33.1
Alam et al (men)	2020	ARIC-NCS	USA	Residents of four communities in the United States	100	47–70	4374	775	24.1
Alam et al (women)	2020	ARIC-NCS	USA	Same as above	0	47–70	6795	1230	24.1
Cao et al (men)	2020	UK biobank	England	Derived from the UK Biobank	100	37–73	213643	1170	8.1
Cao et al (women)	2020	UK biobank	England	Same as above	0	37–73	253337	968	8.1
Chen et al (men)	2021	HABCS	China	Residents of eight longevity areas	100	85.1	3103	486	9
Chen et al (women)	2021	HABCS	China	Same as above	0	85.1	3103	486	9

**Abbreviations**: 3C-Dijon: Three-City Dijon Study; ARIC-NCS: Atherosclerosis risk in communities neurocognitive study; HABCS: Healthy Aging and Biomarkers Cohort Study

**Table 3:** UA detection in included studies.

Author	Year	Sample source	Quantification method of UA	Times of quantification (time)
Euser et al	2009	Serum	Uricase-peroxidase-o-dianisidine	1
Latourte et al	2018	Serum	Colorimetric enzymatic assay	1
Scheepers et al (women)	2019	Serum	Not reported	2
Alam et al (men)	2020	Serum	Uricase enzymatic	1
Alam et al (women)	2020	Serum	Same as above	1
Cao et al (men)	2020	Serum	Uricase enzymatic	1
Cao et al (women)	2020	Serum	Same as above	1
Chen et al (men)	2021	Plasma	Uricase colorimetry	1
Chen et al (women)	2021	Plasma	Same as above	1

**Table 4:** Outcomes and covariates of included studies of UA in relation to risk of cognitive impairment.

Author	Year	Endpoint	Uric acid levels (μmol/L)	RR (95% CI)	Covariates in fully adjusted model
Euser et al	2009	Dementia	1st Quartile	1.0 (reference)	Age, sex, level of education serum creatinine levels, systolic blood pressure, ever smoking, total cholesterol and HDL-Cholesterol levels, diabetes mellitus, waist-to-hip ratio, cardiovascular disease
			2st Quartile	0.95 (0.73–1.23)	
			3st Quartile	0.90 (0.69–1.18)	
			4st Quartile	0.73 (0.55–0.97)	
Latourte et al	2018	Dementia	< 225	1.0 (reference)	BMI, tobacco and alcohol consumption, cholesterol, triglycerides, diabetes mellitus, hypertension, interaction between hypertension and time (age at last follow-up or dementia occurrence), history of cardiovascular disease, glomerular filtration rate, and APOE -Ɛ4, NSAIDs, aspirin or diuretics, C-reactive protein, IL-6 levels, and interaction between IL-6 and time (age at last follow-up or dementia occurrence).
			225–268	1.33 (0.74–2.40)	
			268–315	1.4 (0.75–2.62)	
			≥ 315	2.41 (1.29–4.48)	
Scheepers et al (women)	2019	Dementia	≤ 210	1.0 (reference)	Age, BMI, alcohol consumption, smoking, hypertension, triglycerides, cholesterol, eGFR, socioeconomic status and level of education, and in case of second uric acid value.
			211–270	0.93 (0.69–1.24)	
			≥ 271	0.65 (0.47–0.91)	
Alam et al (men)	2020	Dementia	< 249.9	1.0 (reference)	Age, race-center, sex, education, smoking, total and HDL- cholesterol, waist-to-hip ratio, diabetes status, APOE -Ɛ4, systolic and diastolic blood pressure, diuretic antihypertensive medication use, estimated glomerular filtration rate, western and prudent diet scores, and C-reactive protein
			249.9–301.7	0.96 (0.71–1.29)	
			301.7–354	0.96 (0.71–1.28)	
			≥ 354	0.98 (0.73–1.31)	
Alam et al (women)	2020	Dementia	< 249.9	1.0 (reference)	Age, race-center, sex, education, smoking, total and HDL- cholesterol, waist-to-hip ratio, diabetes status, APOE -Ɛ4, systolic and diastolic blood pressure, diuretic antihypertensive medication use, estimated glomerular filtration rate, western and prudent diet scores, and C-reactive protein
			249.9–301.7	1.10 (0.95–1.27)	
			301.7–354	0.93 (0.78–1.11)	
			≥ 354	1.05 (0.86–1.29)	
Cao et al (men)	2020	Dementia	< 294.55	1.0 (reference)	Townsend deprivation index, income, educational attainment, employment, ethnicity, physical activity, alcohol intake, smoking status, C-reactive protein, total cholesterol, hypertension, diabetes, cardiovascular disease, cancer, long-illness, BMI
			294.55–332.01	0.91 (0.77–1.08)	
			332.01–367.12	0.9 (0.75–1.07)	
			367.12–411.15	0.79 (0.65–0.95)	
			> 411.15	0.8 (0.66–0.96)	
Cao et al (women)	2020	Dementia	< 215.99	1.0 (reference)	Townsend deprivation index, income, educational attainment, employment, ethnicity, physical activity, alcohol intake, smoking status, C-reactive protein, total cholesterol, hypertension, diabetes, cardiovascular disease, cancer, long-illness, BMI
			215.99–248.71	0.91 (0.74–1.12)	
			248.71–280.25	0.82 (0.66–1.02)	
			280.25–321.90	0.76 (0.61–0.94)	
			> 321.90	0.79 (0.64–0.97)	
Chen et al (men)	2021	MCI	< 252.0	1.0 (reference)	Age, sex, education, drinking, smoking, marital status, regular exercise, BMI, central obesity, adequate medical service, hypertension, diabetes mellitus, self-reported history of heart disease, and stroke/ cardiovascular disease
			252.0–305.6	0.89 (0.6–1.34)	
			305.6–365.1	0.45 (0.29–0.71)	
			> 365.1	0.58 (0.37–0.91)	
Chen et al (women)	2021	MCI	< 213.9	1.0 (reference)	Age, sex, education, drinking, smoking, marital status, regular exercise, BMI, central obesity, adequate medical service, hypertension, diabetes mellitus, self-reported history of heart disease, and stroke/ cardiovascular disease
			213.9–265.0	0.82 (0.59–1.14)	
			265.0–320.9	0.96 (0.69–1.34)	
			> 320.9	0.85 (0.59–1.23)	

**Abbreviations**: MCI: Mild cognitive impairment; HDL-Cholesterol: High Density Lipoprotein Cholesterol; BMI: body mass index; APOE -Ɛ4: apolipoprotein E -Ɛ4; NSAIDs: Non-steroidal anti-inflammatory drugs; IL: interleukin; eGFR: estimated glomerular filtration rate.

**Table 5:** Study quality of included studies on serum UA and risk of cognitive decline.

	Selection				Comparability	Outcome			
Reference	Repre-sentative of cases	Selection of controls	Exposure ascertainment	No history of disease	Comparable on confounders	Outcome assessment (by hospital register, medical record or doctors)	Adequate follow-up time (> 5 years)	Follow up rate (> 80%)	Overall quality
Euser et al, 2009	1	1	1	1	2	1	1	0	8
Latourte et al, 2018	0	1	1	1	2	1	1	0	7
Scheepers et al, 2019	0	1	1	1	2	1	1	0	7
Alam et al, 2020	0	1	1	1	2	1	1	0	7
Cao et al, 2020	1	1	1	1	2	1	0	0	7
Chen et al, 2021	0	1	1	1	2	1	1	0	7

### 3.3 Meta-analysis

#### 3.3.1 Relationship between blood UA levels and cognitive impairment

**Fig 2:**
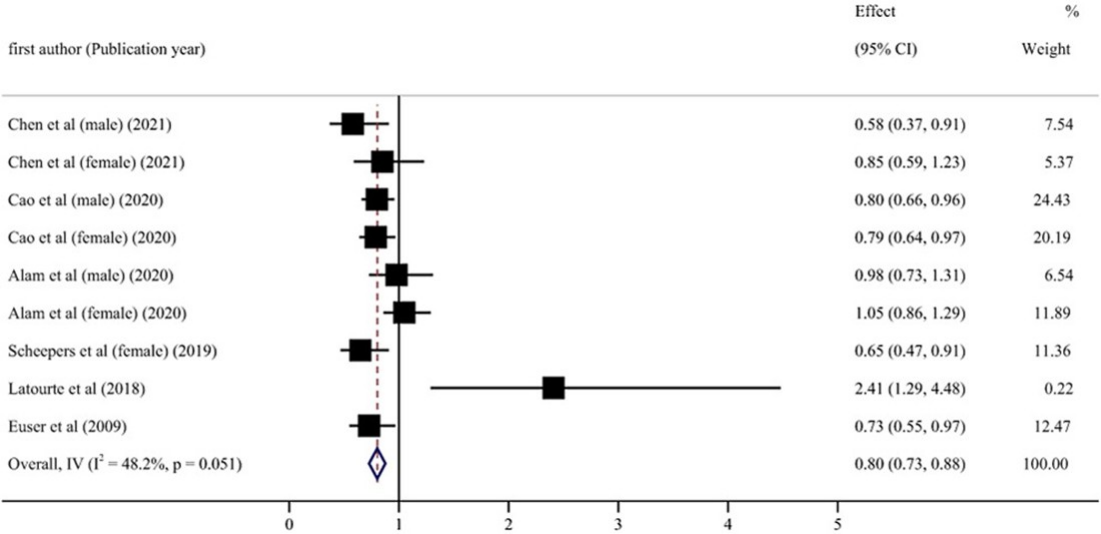
Forest plot of association between highest versus lowest categories of UA and risk of cognitive impairment.

Six articles with nine reports were considered to provide sufficient data for the analysis of blood UA levels and cognitive impairment relationships [30–35]. Compared with the lowest category of blood UA level, the combined RR of cognitive impairment events in the highest group was 0.81 (95% CI: 0.70–0.92, P < 0.001) (Fig 2). Results of the research showed no significant heterogeneity (P = 0.051, I^2^ = 48.2%).

#### 3.3.2 Dose-response analysis

**Fig 3:**
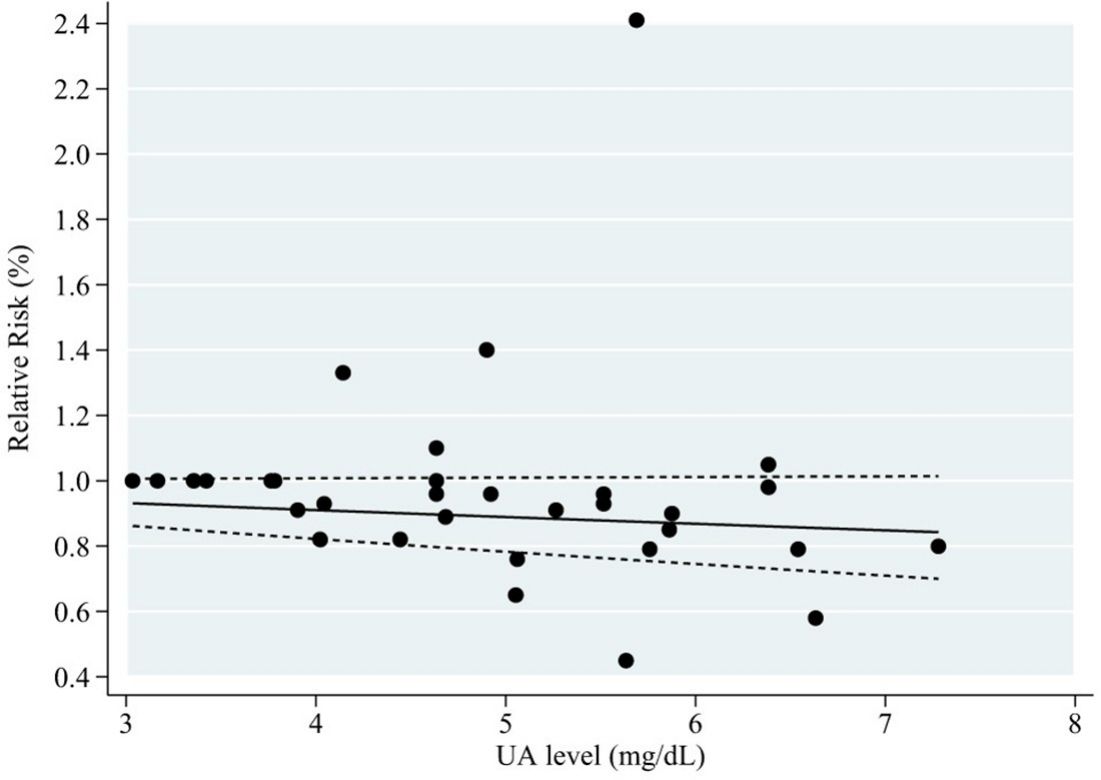
Dose-response analyses of UA levels and risk of cognitive impairment.

**Fig 4:**
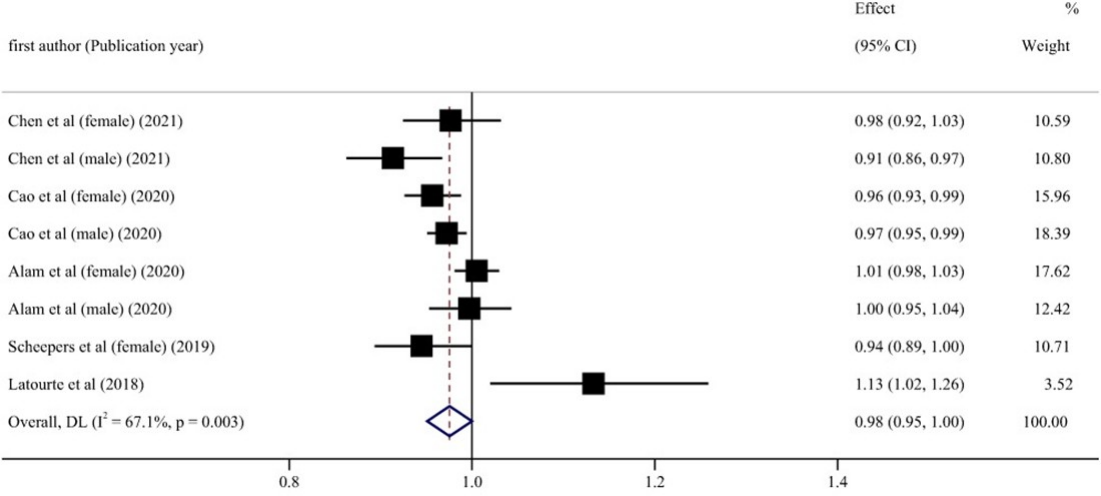
Forest plot of UA levels and risk of cognitive impairment.

**Fig 5:**
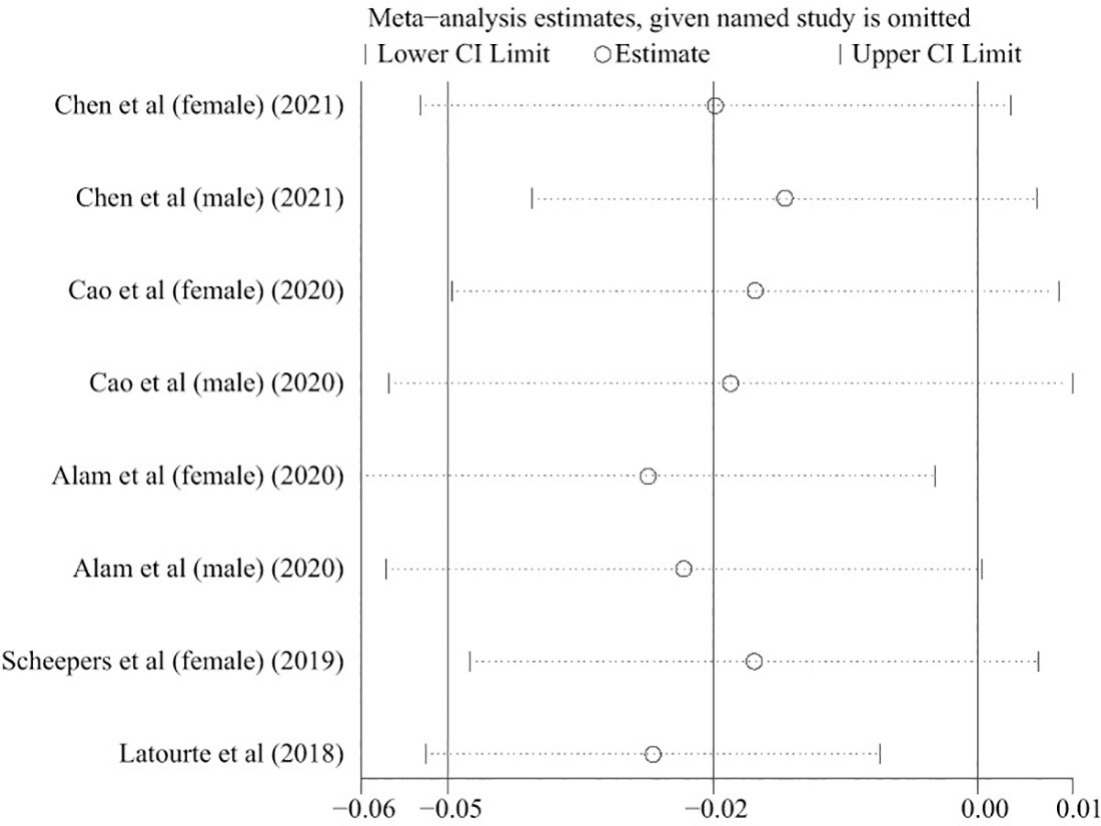
The sensitive plot on the association of UA levels and risk of cognitive impairment.

Five articles with eight reports were considered in this dose-response analysis of blood UA levels and cognitive impairment events [31–35]. UA levels did not appear to be associated with the risk of cognitive impairment in a curvilinear manner (P = 0.51 for nonlinear relationship; Fig 3). The summary RR of cognitive impairment for an increase of 1 mg/dL blood UA level was 0.98 (95% CI: 0.95–1.00; linear trend P = 0.07, I^2^ = 67.1%, heterogeneity P < 0.05) (Fig 4). The regression tests of Begg’s and Egger’s did not demonstrate substantial publication bias (P = 0.902 and P = 0.962). In addition, we conducted a sensitivity analysis to assess whether outcomes were affected by the excluded studies, and found no substantial changes in the results (Fig 5).

#### 3.3.3 Subgroup analysis

**Fig 6:**
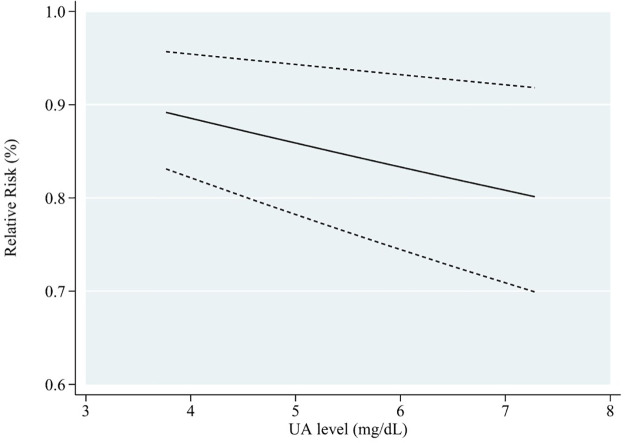
Dose-response plot on the association of UA levels and risk of cognitive impairment in male.

**Fig 7:**
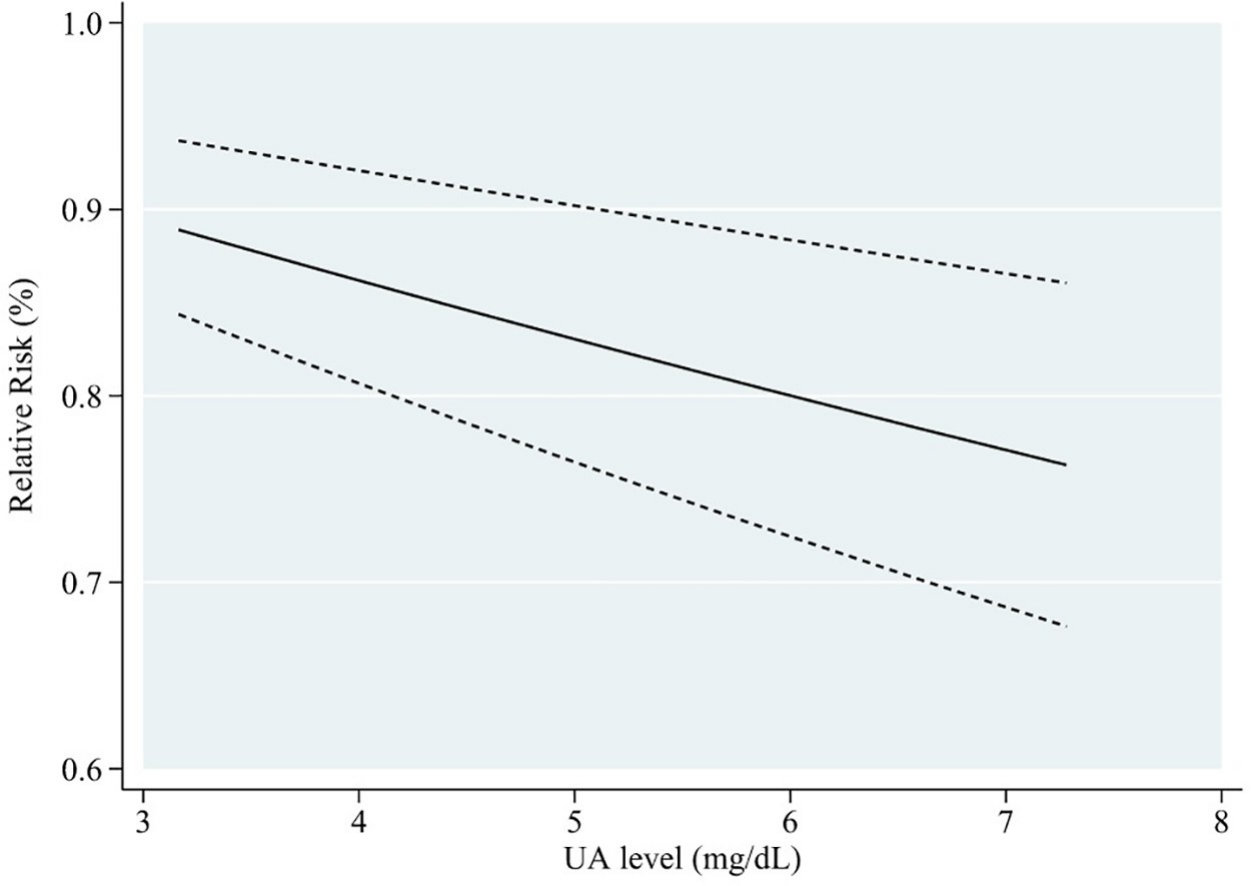
Dose-response plot on the association of UA levels and risk of cognitive impairment with less than 10 years of follow-up.

**Fig 8:**
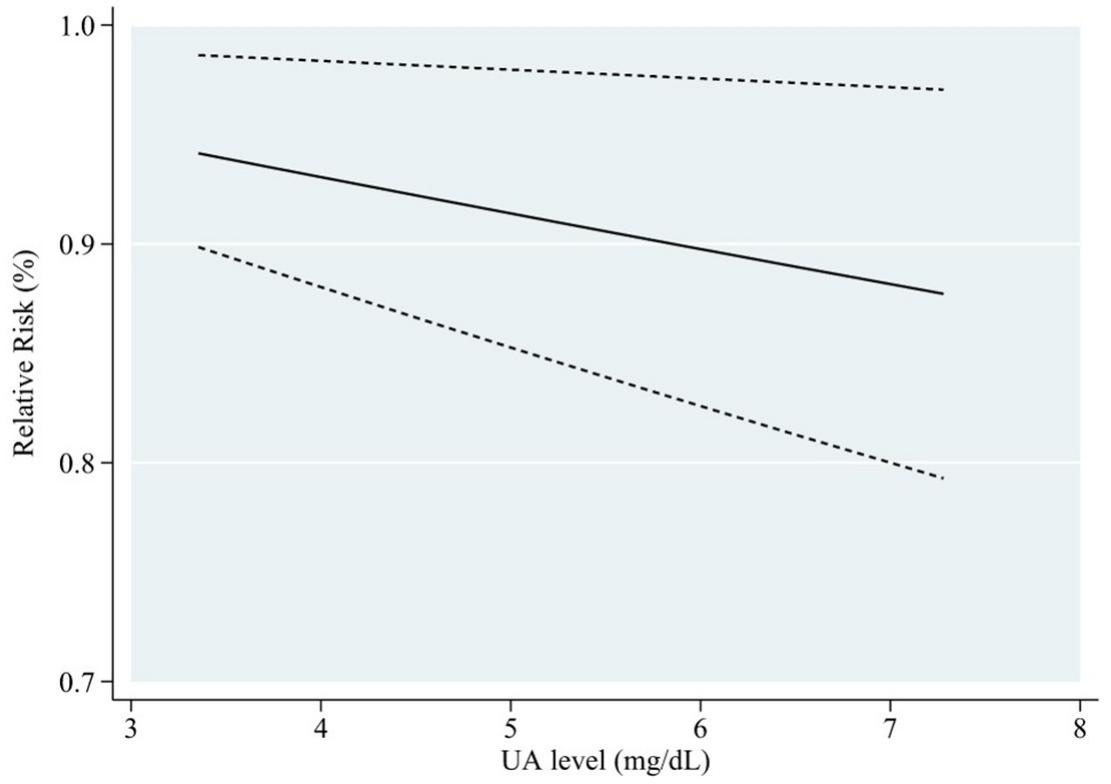
Dose-response plot on the association of UA levels and risk of cognitive impairment with more than 2,000 participants.

**Fig 9:**
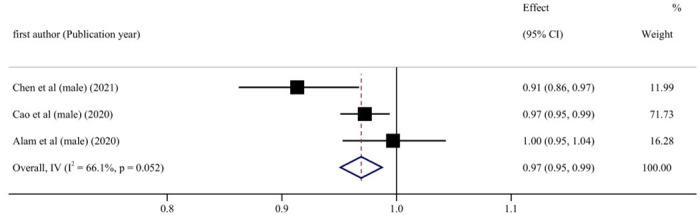
Forest plot of UA levels and risk of cognitive impairment in male.

**Fig 10:**
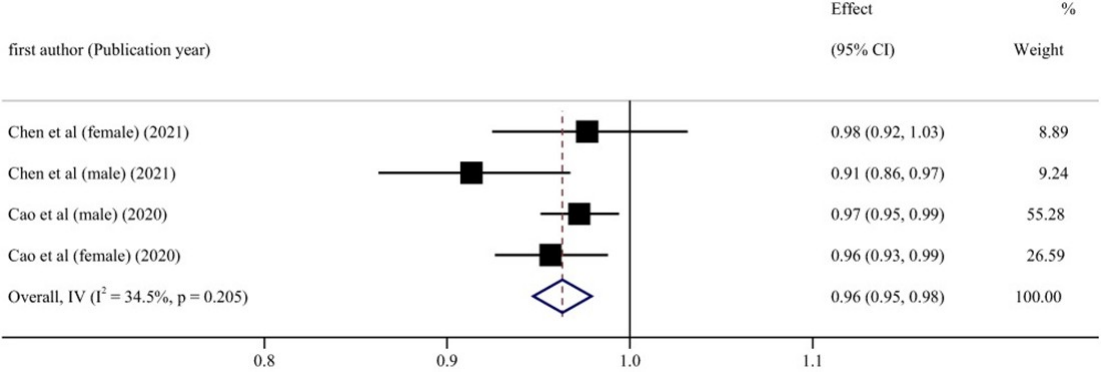
Forest plot of UA levels and risk of cognitive impairment with less than 10 years of follow-up.

**Fig 11:**
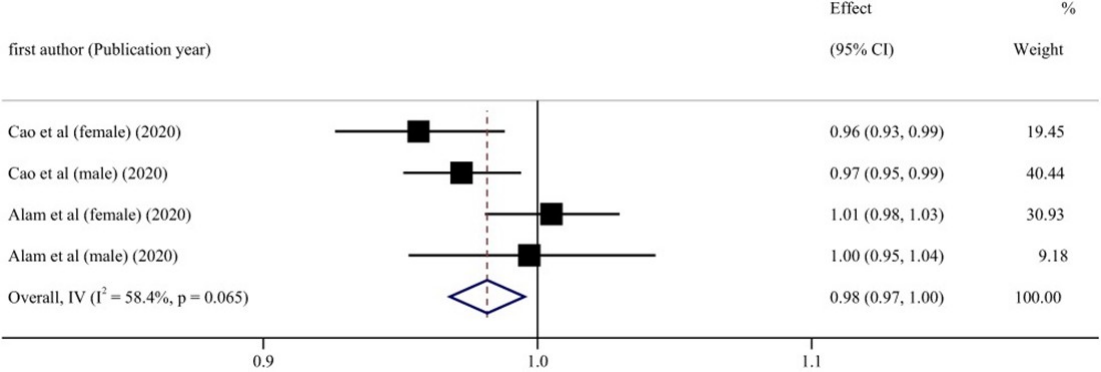
Forest plot of UA levels and risk of cognitive impairment with more than 2,000 participants.

Subgroup analyses were conducted based on gender, age, location of the study, the length of follow-up, dementia type, diabetes status, and number of participants, to explore the potential source of heterogeneity (Table 6). Fig 6 shows that in the male cohort, blood UA levels and cognitive impairment risk exhibited a linear negative relationship (RR = 0.97, 95% CI: 0.95–0.99, P < 0.05). This meta-analysis of blood UA levels found a negatively linear relationship between UA levels and cognitive impairment in individuals who were followed for fewer than ten years (RR = 0.96, 95% CI: 0.95–0.98, P < 0.001) (Fig 7). When stratified according to participants, if the number of participants in total was more than 2000, the incidence risk had a linear negative correlation (RR = 0.98, 95% CI: 0.97–1.00, P < 0.05) (Fig 8). In any of the three subgroup analyses, there was no evidence of significant heterogeneity (P > 0.05) (Figs 9–11).

**Table 6:** An analysis of the relative risk of cognitive impairment based on stratification.

Group	No of reports	Heterogeneity		Model	Meta analysis	
		*P	I^2^(%)		Relative risk (95% CI)	† *P*
Age						
< 60	4	0.247	27.6	Fixed	0.99 (0.98–1.01)	0.593
> 60	4	0.009	74.1	Random	0.97 (0.92–1.03)	0.346
Sex						
Male	3	0.052	66.1	Fixed	0.97 (0.95–0.99)	0.001
Female	4	0.048	62.2	Random	0.97 (0.94–1.01)	0.105
Study location						
European	4	0.033	65.7	Random	0.98 (0.94–1.01)	0.225
Non-European	4	0.019	69.9	Random	0.98 (0.94–1.02)	0.244
Follow-up duration						
≥ 10 years	4	0.028	67.1	Random	1.00 (0.96–1.05)	0.92
< 10 years	4	0.205	34.5	Fixed	0.96 (0.95–0.98)	< 0.001
No of participants						
≥ 2000	4	0.065	58.4	Fixed	0.98 (0.97–1.00)	0.011
< 2000	4	0.008	74.4	Random	0.98 (0.91–1.04)	0.497
Type of dementia						
AD	3	0.131	50.7	Fixed	0.97 (0.95–1.00)	0.088
VaD	3	0.017	75.4	Random	0.98 (0.84–1.14)	0.782
Controlling for diabetes in models						
Yes	7	0.003	69.7	Random	0.98 (0.95–1.00)	0.160
No	1	NA	NA	NA	NA	NA

**Notes**:

*P for heterogeneity;

^†^P for test

**Abbreviations**: CI: confidence interval; VaD: Vascular dementia; AD: Alzheimer’s disease; NA: not analysis

## 4 Discussion

### 4.1 Principal findings

To the best of our knowledge, the first dose-response meta-analysis conducted on prospective cohort studies investigating UA concentrations and cognitive impairment was presented in our study. Based on the nine prospective cohort studies, a significant correlation was identified between blood UA levels and risk of cognitive impairment in the comparison of the highest versus lowest group. The remarkable relationships disappeared when we conducted a dose-response analysis. However, studies including only male participants, with more than 2000 volunteers and followed up for less than 10 years, demonstrated that a reduction in cognitive impairment was associated with higher blood UA levels. Nevertheless, this should be evaluated further.

### 4.2 Results in relation to other studies

Our findings identified that the risk of cognitive impairment is not significantly correlated with blood UA levels. A meta-analysis of 5575 participants pointed out that there is a potential risk of Alzheimer’s disease and Parkinson’s dementia associated with low concentrations of blood UA, but this does not apply to vascular dementia [19]. Another meta-analysis of 1128 cases and 2498 controls found that high UA level in blood was a protective element for Alzheimer’s disease [20]. Different study types, study sample size, cognitive impairment diagnosis, and not well-controlled confounders may have contributed to this discrepancy.

In clinical practice, the normal levels of serum UA range from 2 mg/dL to 7 mg/dL. In hyperuricemia, the serum urate concentration is above 7mg/dL, while in hypouricemia, it is below 2mg/dL [36, 37]. To date, most cohort studies have examined the relationship between UA levels and cognitive trajectories. A national retrospective cohort study of older adults conducted in Korea found that there was a lower risk of dementia among patients with gout compared to the general population, regardless of the type of dementia [38]. This discovery provides lateral support for the beneficial neuroprotective effects of hyperuricemia, which are similar to some of our conclusions. Further study showed that both serum UA baseline levels and dynamic changes may affect cognitive trajectories. Although stable or moderate increases of serum UA levels may benefit cognition, hyperuricemia persisting for a long time can negatively impact cognition [39]. However, the observations of Liu et al. find that both high and low blood UA levels are correlated with an increased risk of cognitive impairment, supporting a U-shaped relationship between these associations [40]. Our study includes the populations with hyperuricemia, normal UA levels and hypouricemia. The included studies lack stratification data of the three populations mentioned above, so we cannot consider them as subgroup variables for further analysis. Future studies may be specifically designed to increase the diversity of the study population to solve this problem.

### 4.3 Interpretation of the results

We further considered the reasons for lack of an overall relationship between blood UA levels and cognitive impairment. First, it is well known that blood UA levels are associated with obesity, alcohol consumption, smoking, physical activity, hypertension, diabetes and other elements [10, 41, 42], that are also risk factors for cognitive impairment. Despite our selection of the most adjusted models, the influence of residual confounding factors on the basis of the results could not be excluded. We lacked an assessment of nutritional status, which might play a partial role in blood UA levels and cognitive function [43]. Studies have also demonstrated that people with low blood UA levels tend to have poorer nutritional status [44, 45], which increases the risk of cognitive impairment [46]. Furthermore, although "one-third" of the total amount of urate in the body comes from dietary purines [47], we cannot ignore the influence of dietary patterns on UA levels. Dietary interventions are considered important for gout prevention [48]. A meta-analysis showed that plant-based dietary patterns were associated with lower UA levels, while animal-based dietary patterns were associated with higher UA levels [49]. Further studies have shown that in populations with normal UA levels, UA concentrations in short- and long-term dieters remain within the normal range even when only plant-based dietary patterns are used [50]. However, due to the wide variation in dietary habits among people born in different countries, geographic regions, and generations, as well as changes in dietary habits during long-term follow-up, most of the included studies lacked data on diet and UA levels, which could be further analyzed in the future by refining the study protocols.

Second, deteriorating health and death are the main hallmarks of the aging process [51]. A potential explanation might be that survival bias reduced the likelihood of people with different levels of blood UA surviving into later life, both inhibiting the development of cognitive impairments and affecting the true assessment of cognitive characteristics in later life. Most of our studies did not consider the character of survival bias in the development of cognitive impairment, which brought bias to the research results.

Third, blood UA levels might modulate cognitive function in a different way. UA has been shown to be a catalyst for human intellectual development has been proven [52], which is due to its anti-oxidant properties and potential neuroprotection [9, 53]. Previous studies have indicated that UA has both oxidation and reduction properties based on differences in the physicochemical environment [11, 54]. UA not only plays a role in oxidative stress as a bioactive pro-inflammatory factor, but also protects neurons under certain conditions and exhibits antioxidant properties [55–57]. Pathological changes in neurodegeneration and cerebrovascular injuries play a synergistic or additive role in the course of cognitive impairment [58, 59]. Several studies, including observational studies and animal experiments, have illustrated that the progression of cognitive impairment is closely linked to oxidative stress and inflammatory responses [59–63], and that blood UA levels are associated with these mechanisms [10, 64–66]. Redox homeostasis is important to maintain normal cellular physiological function [67, 68]. One possible interpretation is that physiological levels of blood UA plays a neuroprotective role in the development of cognitive disorders; however, when UA values are elevated to a certain range and remain high for a long time, the neuroprotective effect might be obscured by the increased vascular risk associated with high blood UA levels.

Fourth, we assessed baseline UA levels and cognitive impairment correlations. Most studies have only measured baseline UA levels, and the role of UA might depend on the duration of its levels. Changes in UA levels occur over the course of the prolonged follow-up [69], and chronically higher UA levels could be associated with cognitive decline [39]. Recently, a community-based cohort study suggested that the interaction between genotype of apolipoprotein E4 and additional genetic risk factors modified the risks of dementia [70]. Similarly, Lee and colleagues found that higher serum UA may interact with apolipoprotein E4 to alleviate cognitive decline in female patients with MCI [71]. The influence of genetic factors in the incidence of cognitive impairment should be further explored.

Furthermore, various cognitive domains may be affected by alterations in UA levels [16, 72]. Huang et al found that low levels (within normal range) of plasma UA had a potentially detrimental effect on cognitive function in the executive domain in adults without hyperuricemia [15]. Yuan et al showed that lower UA levels were associated with poorer performance in situational memory and mental status [73]. A recent cross-sectional study showed that higher UA was associated with poorer performance in the domain of visual memory, but not in the domain of reaction speed [74]. However, some of the studies we included indicated that increased UA levels were correlated with enhanced memory function [30, 35]. We did not explore this further as the included studies were not subdivided regarding the association of UA with different cognitive domains.

Studies in men have shown a significant negative association between blood UA levels and cognitive impairment. Similar to our results, another prospective study suggests that the effect of blood UA levels on spontaneous brain activity and cognitive function varies by gender [75]. Sex hormones may play a vital role in this connection [76]. It is believed that the loss of estrogen plays a role in age-related cognitive changes in neurobiology [77, 78]. Cognitive function of women in a certain age interval may benefit from estrogen supplementation [79]. Higher plasma estrogen levels in women may lead to increased clearance of serum UA by the kidneys, weakening the protective effect of UA on cognition [78, 80]. The specific mechanism needs to be explored further. Our study also shows that the notable relationship vanished when restricted to studies with longer follow-up times. Due to the lack of survival data, a competing risk analysis could not be conducted, which may have biased the results. The power to detect the latent relationship could be increased with a larger sample size. Therefore, an analysis of more than 2000 participants may exhibit remarkable results.

### 4.4 Strengths and limitations

The strengths of the study should be acknowledged. First, a meta-analysis involving prospective cohort studies with long follow-up periods and large sample sizes was conducted, which enabled us to quantitatively evaluate the relationship between blood UA levels and cognitive impairment. Second, we included studies with fully adjusted models to decrease confounding bias. Third, a dose-response analysis was conducted to explore the potential nonlinear relations.

Our findings also need to be interpreted by considering several limitations. First, causality cannot be established, owing to the natural defection of observational studies. Second, although we brought the fully adjusted models into the analysis, the residual confounders cannot be completely ignored. Third, most of the studies provided baseline blood UA levels. However, the concentration of blood UA may change during the long follow-up time. Further reports with repeated blood UA levels are warranted. Lastly, the significant relationship observed in the subgroup analyses should be explained carefully and verified in future studies.

## 5 Conclusion

We found that cognitive impairment did not appear to be correlated with blood UA levels. Higher blood UA levels are related to a decline in cognitive impairment in males. A larger sample size, longer follow-up time and dynamic observation of blood UA levels are needed to confirm these findings. Future directions: 1) Studies should be conducted in hyperuricemia, normal UA levels and hypouricemia populations separately to better quantify the effect of UA levels on cognitive function and to provide guidelines for the management of UA in different populations; 2) To Investigate the effects of UA on different cognitive domains; 3) Large prospective cohort studies with long-term follow-up should be conducted in order to eliminate as much as possible the effects of residual confounders such as dietary patterns, nutritional status and survival data.

## Supporting information

S1 Table PubMed search terms. (DOCX)

S2 Table Embase search terms. (DOCX)

S1 Checklist PRISMA 2009 checklist. (DOCX)

## Abbreviations

CI: Confidence interval
MCI: Mild cognitive impairment
PRISMA: Preferred Reporting Items for Systematic Reviews and Meta-Analyses
RR: Relative risk
UA: Uric acid
WHO: World Health Organization
