# Supplementary material for: Uric acid levels and risk of cognitive impairment: Dose-response meta-analysis of prospective cohort studies
Source: PLoS One. 2023 Nov 2;18(11):e0293832. doi: 10.1371/journal.pone.0293832 (PMC10621826; doi:10.1371/journal.pone.0293832)
Supplement: S1 Table — (DOCX) [file pone.0293832.s001.docx]

**Supporting information**

Supplementary Table 1. PubMed search terms

| Number | Search terms |
| --- | --- |
| #1 | Uric Acid |
| #2 | "Uric Acid"[Mesh] |
| #3 | #1 OR #2 |
| #4 | hyperuricaem* |
| #5 | "Hyperuricemia"[Mesh] |
| #6 | #3 OR #4 |
| #7 | "Gout"[Mesh] |
| #8 | #3 OR #6 OR #7 |
| #9 | cognitive disab* |
| #10 | cognitive decline* |
| #11 | cognitive impairment* |
| #12 | MCI |
| #13 | #9 OR #10 OR #11 OR #12 |
| #14 | "Cognitive Dysfunction"[Mesh] |
| #15 | #13 OR #14 |
| #16 | Alzheimer* |
| #17 | AD |
| #18 | "Alzheimer Disease"[Mesh] |
| #19 | #16 OR #17 OR #18 |
| #20 | Parkinson's dementia |
| #21 | VD |
| #22 | VaD |
| #23 | "Dementia, Vascular"[Mesh] |
| #24 | #21 OR #22 OR #23 |
| #25 | "Dementia, Multi-Infarct"[Mesh] |
| #26 | "Dementia"[Mesh] |
| #27 | "Frontotemporal Dementia"[Mesh] |
| #28 | "AIDS Dementia Complex"[Mesh] |
| #29 | "Lewy Body Disease"[Mesh] |
| #30 | post-stroke cognitive impairment* |
| #31 | PSCI |
| #32 | #30 OR #31 |
| #33 | Cogniti* |
| #34 | "Cognition"[Mesh] |
| #35 | #33 OR #34 |
| #36 | "Cognition Disorders"[Mesh] |
| #37 | #15 OR #19 OR #20 OR #24 OR #25 OR #26 OR #27 OR #28 OR #29 OR #32 OR #35 OR #36 |
| #38 | cohort* |
| #39 | "Cohort Studies"[Mesh] |
| #40 | #38 OR #39 |
| #41 | #8 AND #37 AND #40 |
