# Supplementary material for: Uric acid levels and risk of cognitive impairment: Dose-response meta-analysis of prospective cohort studies
Source: PLoS One. 2023 Nov 2;18(11):e0293832. doi: 10.1371/journal.pone.0293832 (PMC10621826; doi:10.1371/journal.pone.0293832)
Supplement: S2 Table — (DOCX) [file pone.0293832.s002.docx]

**Supporting information**

Supplementary Table 2. Embase search terms

| Number | Search terms |
| --- | --- |
| #1 | 'gout'/exp |
| #2 | 'hyperuricemia'/exp |
| #3 | 'uric acid'/exp |
| #4 | 'uric acid'/exp |
| #5 | 'uric acid' |
| #6 | uric |
| #7 | 'acid'/exp |
| #8 | acid |
| #9 | #7 OR #8 |
| #10 | #6 AND #9 |
| #11 | hyperuricaem* |
| #12 | hyperuricemia |
| #13 | gout |
| #14 | #1 OR #2 OR #3 OR #4 OR #5 OR #10 OR #11 OR #12 OR #13 |
| #15 | cognitive |
| #16 | disab* |
| #17 | #15 AND #16 |
| #18 | cognitive |
| #19 | decline* |
| #20 | #18 AND #19 |
| #21 | cognitive |
| #22 | impairment* |
| #23 | #21 AND #22 |
| #24 | mci |
| #25 | cognitive |
| #26 | dysfunction |
| #27 | #25 AND #26 |
| #28 | alzheimer* |
| #29 | alzheimer |
| #30 | disease |
| #31 | #29 AND #30 |
| #32 | parkinson* |
| #33 | dementia |
| #34 | #32 AND #33 |
| #35 | vd |
| #36 | vad |
| #37 | dementia, |
| #38 | vascular |
| #39 | #37 AND #38 |
| #40 | dementia, |
| #41 | 'multi infarct' |
| #42 | #40 AND #41 |
| #43 | dementia |
| #44 | frontotemporal |
| #45 | dementia |
| #46 | #44 AND #45 |
| #47 | aids |
| #48 | dementia |
| #49 | complex |
| #50 | #47 AND #48 AND #49 |
| #51 | lewy |
| #52 | body |
| #53 | disease |
| #54 | #51 AND #52 AND #53 |
| #55 | 'post stroke' |
| #56 | cognitive |
| #57 | impairment* |
| #58 | #55 AND #56 AND #57 |
| #59 | psci |
| #60 | cogniti* |
| #61 | cognition |
| #62 | cognition |
| #63 | disorders |
| #64 | #62 AND #63 |
| #65 | 'alzheimer disease'/exp |
| #66 | 'multiinfarct dementia'/exp |
| #67 | 'dementia'/exp |
| #68 | 'frontotemporal dementia'/exp |
| #69 | 'hiv associated dementia'/exp |
| #70 | 'diffuse lewy body disease'/exp |
| #71 | 'cognition'/exp |
| #72 | 'cognitive defect'/exp |
| #73 | #17 OR #20 OR #23 OR #24 OR #27 OR #28 OR #31 OR #34 OR #35 OR #36 OR #39 OR #42 OR #43 OR #46 OR #50 OR #54 OR #58 OR #59 OR #60 OR #61 OR #64 OR #65 OR #66 OR #67 OR #68 OR #69 OR #70 OR #71 OR #72 |
| #74 | 'cohort analysis'/exp |
| #75 | cohort* |
| #76 | #74 OR #75 |
| #77 | #14 AND #73 AND #76 |
